# Pathophysiological mechanisms of benign prostatic obstruction in men with small prostate volume and the emerging role of ultra-minimally invasive prostatic urethral dilation: a narrative review

**DOI:** 10.3389/fmolb.2026.1886121

**Published:** 2026-08-05

**Authors:** Kun Shen, Dawei Zhang, Leinan Yu

**Affiliations:** Zhejiang Tongxuan Medical Technology Co., Ltd, Taizhou, Zhejiang, China

**Keywords:** benign prostatic hyperplasia, benign prostatic obstruction, lower urinary tract symptoms, minimally invasive therapy, prostatic urethral dilation, small prostate volume

## Abstract

**Background:**

Lower urinary tract symptoms (LUTS) in men have traditionally been interpreted within a volume-based paradigm, in which benign prostatic enlargement is considered the principal driver of bladder outlet obstruction. However, a subset of patients with an operationally defined small prostate volume (≤30 mL) present with disproportionate symptom severity, indicating that prostate size alone is an insufficient determinant of disease burden.

**Objectives:**

To review the pathophysiological basis of benign prostatic obstruction in men with small prostate volume and to evaluate the emerging role of prostatic urethral dilation (PUD) within a pathophysiology-informed therapeutic framework.

**Evidence Synthesis:**

Small-volume BPO is increasingly recognized as a functionally driven condition involving multiple interacting processes. Enhanced α_1_-adrenergic–mediated smooth muscle tone contributes to dynamic obstruction, while chronic inflammation promotes extracellular matrix remodeling and fibrotic stiffening, leading to reduced urethral compliance. In parallel, alterations in neural signaling within the bladder–prostate axis may amplify both storage and voiding symptoms. These mechanisms collectively disrupt the relationship between prostate size and symptom severity. Current treatments predominantly target either smooth muscle tone or anatomical obstruction but have limited impact on fibrosis, tissue biomechanics, or neural dysregulation. PUD represents a non-ablative, non-resective intervention that reduces urethral resistance through controlled mechanical expansion while preserving mucosal integrity. This resistance-modulating approach may be pathophysiologically relevant to small-volume BPO. Early clinical studies have reported improvements in urinary flow and symptom scores, although the evidence base remains limited.

**Conclusion:**

Small-volume BPO should be considered a distinct clinical phenotype in which functional mechanisms play a prominent role, although anatomical contributors may still coexist in selected patients. PUD may represent an emerging dilation-based strategy that is potentially relevant in this setting. High-quality randomized trials with long-term follow-up are required to define its durability, safety, and position in contemporary management algorithms.

## 1 Introduction

Benign prostatic hyperplasia (BPH) represents a major contributor to lower urinary tract symptoms (LUTS) in aging men and remains a significant source of morbidity worldwide (Roehrborn, 2005). The prevalence of histological BPH increases markedly with age, affecting over half of men older than 60 years and the majority of those beyond 80 years (Roehrborn, 2005). In parallel with population aging, the clinical and economic burden associated with BPH-related LUTS continues to grow. In addition to age-related hormonal changes, metabolic conditions such as obesity, type 2 diabetes, and metabolic syndrome have been increasingly recognized as important contributors to disease development, underscoring the multifactorial nature of BPH (Hughes et al., 2023).

For the purpose of this review, the terminology related to prostate-associated male LUTS requires clear separation (Roehrborn, 2005; European Association of Urology, 2026). BPH is used to describe benign histological changes within the prostate, mainly involving stromal and epithelial proliferation (Roehrborn, 2005). BPE refers to an increase in prostate size detected clinically or by imaging, but enlargement itself does not necessarily indicate clinically relevant obstruction. By contrast, BPO denotes obstruction at the bladder outlet that is attributable to the prostate and is usually assessed through clinical symptoms, uroflowmetry, post-void residual urine, imaging findings, and, when necessary, pressure-flow studies (Abrams et al., 2002; European Association of Urology, 2026). Thus, although BPH, BPE, and BPO are closely related in clinical practice, they describe different aspects of the disease process. The present review therefore focuses on small-volume BPO or clinically suspected prostate-related outlet obstruction, rather than on prostate enlargement alone.

Conventional understanding of male LUTS has largely been based on a volume-driven model, in which benign prostatic enlargement (BPE) leads to benign prostatic obstruction (BPO). Accordingly, established treatments have focused on reducing prostate volume or removing obstructive tissue, including pharmacological therapies such as α_1_-adrenergic receptor antagonists and 5α-reductase inhibitors (5-ARIs), as well as surgical procedures such as transurethral resection of the prostate (TURP) (Miernik and Gratzke, 2020). However, this framework does not adequately account for the wide variability in symptom severity observed in clinical practice.

A clinically relevant subgroup of men experiences moderate-to-severe LUTS despite having a prostate volume within the lower range, commonly operationalized as ≤30 mL in clinical studies and treatment algorithms. This threshold should be interpreted as a pragmatic clinical boundary rather than a strict biological demarcation. Its use is supported by guideline-based size stratification, in which smaller prostates are commonly distinguished from glands more suitable for tissue-resective procedures. For example, transurethral incision of the prostate is generally considered for prostates below 30 mL in selected patients, whereas transurethral resection of the prostate is commonly applied to intermediate-sized glands. In addition, the rationale for volume-reducing medication becomes more evident as prostate size increases, further supporting the distinction between small-volume and enlargement-dominant phenotypes. On this basis, the ≤30 mL cutoff provides a clinically useful framework for identifying patients in whom prostate size alone may be insufficient to explain symptom severity or outlet resistance (Hughes et al., 2023; Cornu et al., 2023; Xu et al., 2025).

Emerging data further support the concept that LUTS is not solely a prostate-driven condition but rather a system-level disorder involving coordinated interactions between the prostate, bladder, urothelium, and neural pathways (Elliott and Payne, 2012). Within this framework, the “bladder–prostate axis” provides a more comprehensive explanation for symptom heterogeneity and treatment response. Consequently, reliance on prostate volume alone as a surrogate for disease severity or therapeutic decision-making appears increasingly inadequate.

These considerations have important implications for clinical management. Treatments designed primarily to reduce prostate size or eliminate obstructive tissue may not effectively address the underlying functional and microenvironmental abnormalities in all patients, particularly those with small prostate volume. This mismatch highlights the need for a shift toward a mechanism-based, phenotype-oriented treatment strategy, in which therapeutic selection is guided by dominant pathophysiological processes rather than anatomical features alone (Franco et al., 2023; Abt et al., 2021).

In this context, non-resective and function-preserving interventions have gained increasing attention. Prostatic urethral dilation (PUD) represents an emerging approach that reduces urethral resistance through controlled mechanical expansion without tissue ablation. Unlike conventional resective or ablative techniques, this strategy preserves mucosal integrity and minimizes structural injury. From a mechanistic perspective, PUD may be relevant to selected patients with small prostate volume, in whom functional obstruction rather than tissue bulk may predominate.

Despite growing clinical interest, the role of PUD remains incompletely defined. Existing evidence is largely derived from early-stage clinical studies and observational cohorts, and its long-term efficacy, durability, and comparative effectiveness relative to established therapies remain to be established (Roehrborn, 2005; Miernik and Gratzke, 2020). In this review, small prostate volume is defined as a prostate volume of ≤30 mL, and the term small-volume BPO is used when bladder outlet obstruction is confirmed or clinically suspected in this anatomical setting.

## 2 Methods

This narrative review was based on a structured literature search and selective synthesis of published evidence (Green et al., 2006; Grant and Booth, 2009). Literature was identified through searches of PubMed, Web of Science, Google Scholar, CNKI, and Wanfang Data from database inception to June 2026. The search strategy combined terms related to benign prostatic hyperplasia, benign prostatic enlargement, benign prostatic obstruction, small prostate volume, lower urinary tract symptoms, bladder outlet obstruction, functional obstruction, smooth muscle tone, prostatic fibrosis, extracellular matrix remodeling, chronic inflammation, oxidative stress, neural dysregulation, detrusor overactivity, detrusor underactivity, urodynamics, minimally invasive surgical therapies, and prostatic urethral dilation. Reference lists of relevant reviews, clinical studies, guidelines, and consensus documents were also manually screened to identify additional sources.

Studies were considered relevant if they addressed the pathophysiology, clinical evaluation, or treatment of male LUTS/BPH/BPO, particularly in relation to small prostate volume, functional obstruction, tissue remodeling, bladder–prostate interaction, or minimally invasive treatment strategies. Original clinical studies, mechanistic studies, guideline documents, expert consensus reports, and high-quality review articles were included when they contributed directly to the scope of this review. Articles were excluded if they were unrelated to male LUTS/BPH/BPO, lacked sufficient methodological or clinical relevance, or did not provide information applicable to the mechanisms or therapeutic questions discussed here. Both English-language and Chinese-language publications were considered when relevant, particularly for emerging evidence on prostatic urethral dilation.

Because this article is a narrative review rather than a systematic review or meta-analysis, formal risk-of-bias assessment and quantitative evidence synthesis were not performed. The final selection of references was made by the authors based on relevance to the review objectives, methodological quality, clinical importance, and contribution to a balanced discussion of established therapies, emerging interventions, and evidence limitations.

## 3 Pathophysiological mechanisms of BPO in men with small prostate volume

### 3.1 Functional obstruction and smooth muscle tone

In patients with small prostate volume, dynamic factors often make a substantial contribution to bladder outlet resistance, particularly when glandular enlargement is limited. Increased smooth muscle tone within the prostatic stroma, periurethral tissue, and bladder neck can elevate urethral resistance even in the absence of marked prostate enlargement. However, this functional component should not be interpreted as excluding anatomical contributors. Bladder neck configuration, periurethral narrowing, median lobe morphology, and fibrotic stiffening may still contribute to obstruction in selected patients (Miernik and Gratzke, 2020; Murad et al., 2023).

α_1_-adrenergic receptors, particularly the α_1_A subtype, are abundantly expressed in prostatic tissue and regulate smooth muscle contraction through calcium-dependent signaling pathways (Murad et al., 2023; Yoosuf et al., 2024). Persistent sympathetic activation, accompanied by elevated norepinephrine release, can sustain a high-contractile state, thereby maintaining functional obstruction even in the absence of significant anatomical narrowing (Yoosuf et al., 2024).

This dynamic mechanism may be particularly relevant in men with small prostate volume because the static component generated by adenomatous bulk is relatively limited (Murad et al., 2023; Miernik and Gratzke, 2020). In larger prostates, gland enlargement and tissue compression may constitute a substantial part of bladder outlet resistance. By contrast, when prostate size is small, changes in prostatic stromal tone, periurethral contraction, and bladder neck tension may account for a larger proportion of the total resistance encountered during voiding. Therefore, even a moderate increase in α_1_-adrenergic-mediated smooth muscle contraction may produce clinically meaningful impairment of urinary flow in this subgroup (Yoosuf et al., 2024). This interpretation does not exclude anatomical contributors but suggests that the balance between dynamic and static components may shift toward dynamic obstruction in many patients with small-volume BPO (Bushman and Jerde, 2016; Murad et al., 2023).

Clinically, this dynamic component is reflected in urodynamic findings, including increased urethral resistance and reduced maximum urinary flow rate (Qmax) (Bushman and Jerde, 2016). These observations support the view that smooth muscle tone is a major, although not exclusive, determinant of outlet resistance in selected patients with small prostate volume.

### 3.2 Fibrosis and extracellular matrix remodeling

Beyond dynamic obstruction, structural alterations in the extracellular matrix (ECM) represent an important determinant of urethral mechanics in small-volume BPO. Under physiological conditions, the prostatic stroma maintains a balance between smooth muscle and connective tissue, allowing adequate elasticity and compliance.

During disease progression, excessive deposition of collagen types I and III, along with disruption of elastic fibers, leads to increased tissue stiffness and reduced compliance (Khan et al., 2025; Fibbi et al., 2010). Transforming growth factor-β (TGF-β) signaling is a key mediator of fibroblast activation and collagen synthesis, driving fibrotic remodeling (Khan et al., 2025).

Reduced tissue compliance limits urethral distensibility during voiding, thereby increasing outlet resistance independently of luminal narrowing. Moreover, fibrosis may diminish responsiveness to α_1_-adrenergic receptor antagonists, suggesting that structural remodeling can directly influence treatment efficacy (Ren et al., 2024).

### 3.3 Chronic inflammation and microenvironmental changes

Chronic inflammation is increasingly recognized as an active contributor to structural and functional changes in BPH/BPO rather than merely an associated histological finding (Fibbi et al., 2010; Bushman and Jerde, 2016). Inflammatory cell infiltration within prostatic tissue can induce the release of cytokines and growth factors, including interleukin-6 (IL-6), tumor necrosis factor-α (TNF-α), and transforming growth factor-β (TGF-β). These mediators may stimulate fibroblast activation, extracellular matrix deposition, and stromal remodeling, thereby increasing tissue stiffness and reducing urethral compliance (Fibbi et al., 2010; Khan et al., 2025).

The prostatic microenvironment in small-volume BPO involves interactions among epithelial cells, stromal cells, immune cells, extracellular matrix components, vascular structures, and neural elements. Under persistent inflammatory stimulation, this local environment may shift toward a remodeling-prone state, characterized by altered cellular signaling, increased matrix turnover, and impaired tissue elasticity. These changes can increase outlet resistance even when prostate enlargement is not prominent (Xu et al., 2025; Bushman and Jerde, 2016).

Oxidative stress and local hypoxia may further amplify this process. Reactive oxygen species generated during chronic inflammation can aggravate cellular injury, promote collagen accumulation, and interfere with normal repair responses. In parallel, microvascular dysfunction and reduced tissue perfusion may contribute to ischemia-related changes in smooth muscle contractility and tissue biomechanics. Together, these mechanisms link chronic inflammation to both fibrotic remodeling and functional obstruction (Kaltsas et al., 2025; Khan et al., 2025).

Inflammatory signaling may also influence neural regulation by sensitizing afferent pathways and modifying neurotransmitter release. Through this neuro-inflammatory interaction, local prostatic inflammation may contribute not only to structural remodeling but also to urgency, frequency, nocturia, and heightened symptom perception. Therefore, inflammation, fibrosis, oxidative stress, microvascular dysfunction, and neural dysregulation should be viewed as interconnected components of a shared pathological network in small-volume BPO (Savvides et al., 2024).

### 3.4 Neural dysregulation and the bladder–prostate axis

In the present review, the bladder–prostate axis is used as an integrative concept that includes neural signaling, inflammatory crosstalk, bladder remodeling, and urothelial sensory dysfunction. It therefore refers to a combined functional network rather than a single anatomical pathway. Neural regulation within the bladder–prostate axis plays a crucial role in coordinating urinary function (Savvides et al., 2024). Alterations in this system may lower the sensory threshold of the bladder, contributing to urgency and increased frequency, while enhanced sympathetic efferent activity promotes smooth muscle contraction and elevates outlet resistance (Yoosuf et al., 2024).

At the peripheral level, this sensory component may involve sensitization of bladder and pelvic afferent pathways. Suburothelial Aδ- and C-fiber afferents respond to bladder filling, chemical irritation, and inflammatory mediators (Fowler et al., 2008; de Groat et al., 2015). Under inflammatory conditions, ion channels such as transient receptor potential vanilloid 1 (TRPV1) may become more responsive, thereby lowering the threshold for afferent firing. Sensory neuropeptides, including substance P and calcitonin gene-related peptide (CGRP), may further contribute to neurogenic inflammation by promoting local vascular changes, immune cell activation, and crosstalk between sensory nerves and stromal or urothelial cells. In this context, inflammatory changes in the prostate or periurethral region may amplify bladder sensation even when anatomical enlargement is limited.

Central mechanisms may also contribute, although the evidence in small-volume BPO remains indirect. Persistent peripheral input from the prostate, bladder neck, or bladder wall may contribute to altered excitability within spinal micturition circuits and supraspinal regulatory pathways (Fowler et al., 2008). This process may help explain why some patients show disproportionate urgency, frequency, nocturia, or symptom perception relative to prostate size. However, direct evidence linking TRPV1 activation, substance P, CGRP, or central sensitization specifically to small-volume BPO is still limited (Chen et al., 2024; Li et al., 2021; Devesa et al., 2014). Therefore, neural dysregulation should be regarded as a plausible but less well-established component of the proposed framework compared with smooth muscle tone, inflammation, and fibrosis.

The bladder component of this axis is also important in the small-volume phenotype. Detrusor overactivity may cause urgency, frequency, and nocturia even when outlet resistance is only mild, whereas impaired bladder compliance can increase storage pressure and worsen symptom perception (Chess-Williams and Sellers, 2023; Abt et al., 2021; European Association of Urology, 2026). Conversely, detrusor underactivity may present with weak stream, incomplete emptying, or increased post-void residual urine, thereby mimicking or coexisting with prostate-related obstruction (Abrams et al., 2002; European Association of Urology, 2026). These bladder-side abnormalities may arise independently, but they may also develop secondary to long-standing outlet resistance through bladder wall remodeling and altered detrusor contractility. For this reason, pressure-flow studies combined with cystometric assessment may be useful in selected patients, particularly when symptoms are disproportionate to prostate size or when the relative contribution of prostatic obstruction and primary bladder dysfunction is uncertain (Abrams et al., 2002; European Association of Urology, 2026).

**FIGURE 1:**
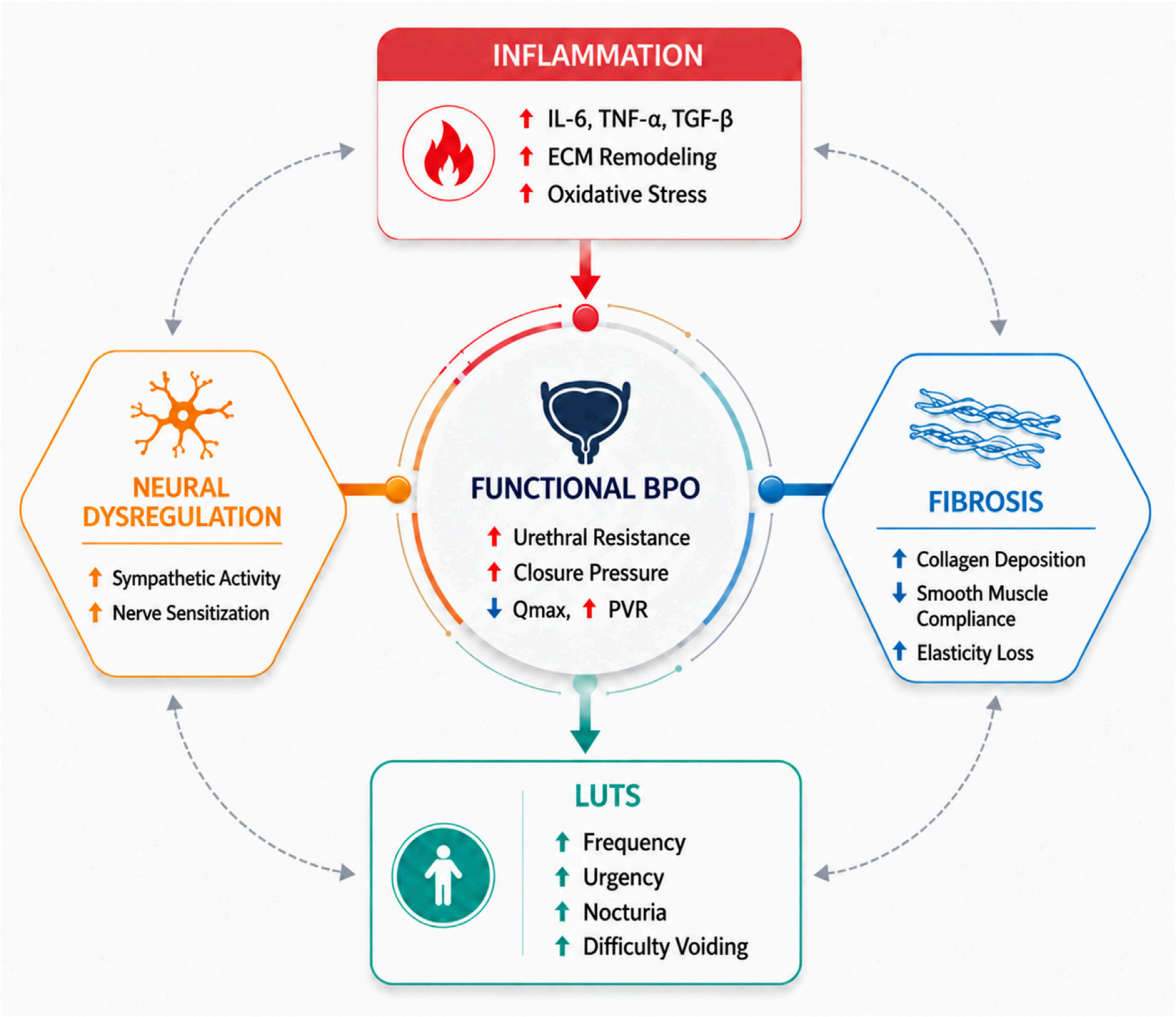
Circular medical diagram illustrating the interrelationships between inflammation, neural dysregulation, fibrosis, benign prostatic obstruction (BPO), and lower urinary tract symptoms (LUTS), with detailed arrows indicating specific contributing factors and outcomes for each component. Abbreviations: BPO, benign prostatic obstruction; LUTS, lower urinary tract symptoms; Qmax, maximum urinary flow rate; PVR, post-void residual volume; ECM, extracellular matrix; IL-6, interleukin-6; TNF-α, tumor necrosis factor-α; TGF-β, transforming growth factor-β; ROS, reactive oxygen species.

Collectively, these mechanisms form an interconnected network rather than acting in isolation. Chronic inflammation promotes fibrotic remodeling, fibrosis alters tissue biomechanics and reduces urethral compliance, while neural dysregulation amplifies both smooth muscle tone and symptom perception (Bushman and Jerde, 2016; Xu et al., 2025). This integrative framework provides a more comprehensive explanation for the dissociation between prostate size and symptom severity in small-volume BPO, as illustrated in Figure 1.

This figure presents a conceptual synthesis of potential mechanisms involved in small-volume BPO rather than direct evidence that each pathway is present in every patient or is modified by any specific intervention (Bushman and Jerde, 2016; Xu et al., 2025). In men with small prostate volume and confirmed or clinically suspected BPO, LUTS may result from interacting functional and microenvironmental mechanisms, while anatomical contributors may still be present in selected patients. Chronic inflammation may promote oxidative stress, extracellular matrix remodeling, stromal fibrosis, and neural sensitization. Fibrotic remodeling can reduce urethral compliance and tissue elasticity, whereas sympathetic overactivity may increase smooth muscle contractility and outlet resistance. The interaction among inflammation, neural dysregulation, and altered tissue biomechanics may form a self-reinforcing pathological loop, contributing to reduced maximum urinary flow rate, increased post-void residual urine, impaired voiding function, and persistent LUTS (de Groat et al., 2015).

## 4 Limitations of current treatment strategies

Current treatment strategies for benign prostatic obstruction (BPO) include medical therapy, conventional surgery, and minimally invasive surgical therapies (MISTs). Although these approaches are effective in many patients, their limitations become more evident in men with small prostate volume, in whom functional, inflammatory, and microenvironmental abnormalities may contribute more substantially to symptoms than prostate enlargement itself (Miernik and Gratzke, 2020; McVary et al., 2011).

Medical therapy remains the first-line option for most patients with LUTS/BPO. α_1_-adrenergic receptor antagonists reduce smooth muscle tone and improve the dynamic component of obstruction by lowering urethral resistance. However, their effects are largely limited to functional smooth muscle relaxation and do not substantially reverse fibrosis, extracellular matrix remodeling, chronic inflammation, or altered tissue biomechanics (Miernik and Gratzke, 2020; McVary et al., 2011). This may partly explain why symptom improvement is incomplete in some patients or gradually decreases over time. In addition, adverse effects such as dizziness, orthostatic hypotension, fatigue, and ejaculatory dysfunction may reduce long-term adherence, particularly in elderly individuals and sexually active patients (Plochocki and King, 2022).

5-ARIs primarily target androgen-dependent prostate growth and are generally more effective in patients with enlarged prostates. In small-volume BPO, however, the contribution of gland enlargement to outlet resistance appears relatively limited, which may explain the less pronounced therapeutic benefit observed in this subgroup (Ren et al., 2024). Their delayed onset of action and potential adverse effects on sexual function, including reduced libido and erectile dysfunction, may further affect patient acceptance and treatment persistence (Plochocki and King, 2022).

Conventional surgical procedures such as transurethral resection of the prostate (TURP) and holmium laser enucleation of the prostate (HoLEP) remain the standard interventions for relieving benign prostatic obstruction. By removing obstructive tissue, these procedures generally provide durable improvements in urinary flow and symptom scores. Nevertheless, their therapeutic principle is fundamentally based on tissue resection. As a result, they may not adequately address functional obstruction, chronic inflammation, fibrosis, or neural dysregulation, all of which may contribute significantly to symptom generation in small-volume BPO (Miernik and Gratzke, 2020; Abt et al., 2021). In addition, these procedures are associated with recognized complications, including bleeding, retrograde ejaculation, urinary incontinence, urethral stricture, and perioperative morbidity (Abt et al., 2021). Preservation of sexual and continence function has therefore become an increasingly important consideration in contemporary treatment selection, especially among younger and sexually active patients (Couteau et al., 2021).

Several minimally invasive surgical therapies (MISTs) have been developed to reduce perioperative morbidity while preserving functional outcomes (Franco et al., 2023; Abt et al., 2021). Prostatic urethral lift (PUL) mechanically retracts prostatic tissue to widen the urethral lumen and can provide relatively rapid symptom relief with limited impact on sexual function. However, its efficacy may be reduced in patients with specific anatomical features, such as a prominent median lobe, and most available studies have involved patients with moderate prostate enlargement (Franco et al., 2023).

Water vapor thermal therapy (Rezūm) improves symptoms through targeted thermal ablation, leading to gradual tissue necrosis and volume reduction. Although effective in selected patients, the procedure still depends on tissue destruction and may be associated with postoperative irritative symptoms and delayed clinical improvement (Das et al., 2020).

Similarly, prostatic artery embolization (PAE) reduces prostate volume by inducing ischemic changes through selective arterial occlusion. While less invasive than conventional surgery, clinical outcomes remain variable, and concerns persist regarding technical complexity, radiation exposure, retreatment rates, and long-term durability (Abt et al., 2021).

Temporary implantable devices such as the temporarily implanted nitinol device (iTIND) aim to remodel the prostatic urethra without tissue removal. Early clinical results are encouraging. However, long-term evidence remains limited, and optimal patient selection criteria have yet to be clearly defined (Das et al., 2020).

Taken together, most currently available therapies continue to focus on tissue reduction, ablation, ischemia, or mechanical displacement. This treatment logic may be less suitable for patients with small prostate volume, in whom functional obstruction, fibrosis, altered tissue biomechanics, and microenvironmental remodeling appear to play a more central role than gland size alone. These limitations highlight the need for mechanism-oriented and tissue-preserving therapeutic strategies capable of directly targeting urethral resistance while minimizing structural injury and functional impairment (Franco et al., 2023).

## 5 Emerging role of prostatic urethral dilation: mechanism, current evidence, and limitations

**FIGURE 2:**
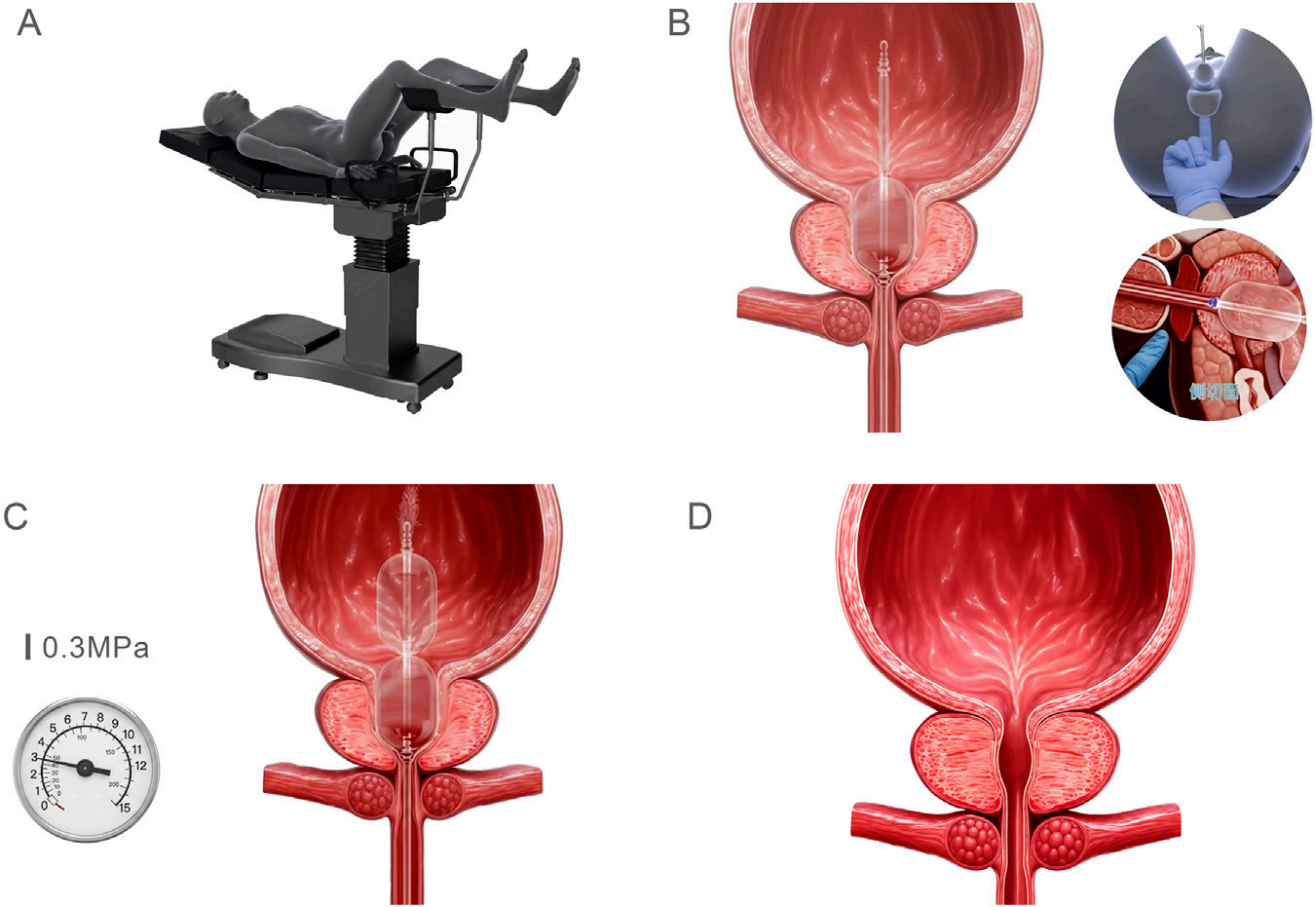
Four-panel medical illustration showing the procedural steps of prostatic urethral dilation (PUD): **(A)** Patient in lithotomy position on a surgical table; **(B)** After confirming the position of the localization prominence by transperineal palpation, the surgeon first inflated the posterior balloon; **(C)** Cross-section of bladder and prostate with an inflated balloon catheter inflated and pressure gauge; **(D)** Cross-section of bladder and prostate after PUD. This schematic was adapted from an animation video produced and owned by Zhejiang Tongxuan Medical Technology Co., Ltd., with permission from the copyright holder.

PUD is a non-resective approach designed to reduce urethral resistance through controlled mechanical expansion of the prostatic urethra. In this review, the term “ultra-minimally invasive” is used descriptively to denote the non-resective and non-ablative nature of the procedure, rather than to indicate a separate regulatory category or proven superiority over other minimally invasive surgical therapies. Recent technical developments, including dual-balloon catheter systems and improved biomaterials, have been reported to enhance procedural stability and positioning accuracy (Huang et al., 2025; Shao et al., 2025). The key procedural steps of PUD, including patient positioning, localization of the catheter, controlled water-filled balloon dilation, and assessment of the dilation outcome, are illustrated in Figure 2.

Unlike conventional resective or ablative procedures, PUD enlarges the urethral lumen without tissue removal or thermal injury. Preservation of urethral mucosal integrity may help reduce direct structural damage and potentially limit secondary inflammatory responses and fibrotic remodeling (Fibbi et al., 2010; Kaltsas et al., 2025). The biological basis for a sustained reduction in urethral resistance after dilation has not yet been fully clarified (Huang et al., 2025). One possible explanation is that radial expansion may enlarge the prostatic urethral lumen while loosening stiff periurethral fibromuscular constraints. In patients with reduced urethral compliance, this process may redistribute mechanical load across collagen-rich stromal tissue and partially reduce the restrictive effect of localized fibrotic remodeling (Bushman and Jerde, 2016). Stretch applied to periurethral smooth muscle may also alter short-term contractile responsiveness or mechanosensitive signaling, although the persistence of such effects remains uncertain. In addition, changes in local sensory input after dilation may contribute to altered outlet perception or voiding dynamics, but this possibility is still largely hypothetical (de Groat et al., 2015). Therefore, PUD should be viewed as a dilation-based intervention with a plausible mechanical rationale, rather than as a proven modifier of fibrosis, inflammation, or neural regulation. Accordingly, any proposed effects of PUD on urethral compliance, periurethral fibrosis, inflammatory activity, or neural signaling should be interpreted as hypotheses requiring direct translational validation, rather than as established clinical mechanisms (Murad et al., 2023; Xu et al., 2025; Huang et al., 2025). This characteristic may be particularly relevant in small-volume BPO, where functional obstruction and microenvironmental alterations appear to contribute more substantially to symptoms than prostate enlargement alone (Murad et al., 2023; Xu et al., 2025).

Preliminary clinical studies have reported improvements in maximum urinary flow rate (Qmax), post-void residual volume, and LUTS following dilation-based treatment (Shao et al., 2025). However, current evidence remains limited and is derived mainly from early clinical experience and observational studies. Compared with conventional surgical procedures such as TURP and HoLEP, non-resective approaches may also be relevant when preservation of sexual and ejaculatory function is an important treatment consideration (Couteau et al., 2021), which has become an increasingly important consideration in patient-centered treatment selection.

Within the broader landscape of minimally invasive therapies, PUD differs procedurally and biomechanically from existing approaches. PUL primarily relies on mechanical retraction of prostatic tissue, whereas therapies such as Rezūm and PAE achieve symptom relief through tissue necrosis and subsequent volume reduction (Das et al., 2020; Murad et al., 2023). In contrast, PUD attempts to reduce urethral resistance through controlled expansion while preserving tissue viability. This distinction may be relevant in selected patients with small prostate volume, in whom functional obstruction may contribute disproportionately to outlet resistance when tissue bulk is limited (Murad et al., 2023).

From a conceptual perspective, PUD differs from tissue-removing or tissue-ablating procedures because it attempts to reduce outlet resistance through temporary circumferential expansion rather than resection, thermal injury, ischemia, or permanent tissue retraction. This rationale may be relevant in small-volume BPO, particularly when reduced urethral compliance and dynamic outlet resistance are suspected to contribute to symptoms. However, this interpretation should remain cautious. At present, direct evidence that PUD produces durable changes in urethral compliance, reverses stromal fibrosis, or modifies inflammatory or neural pathways is lacking (Huang et al., 2025; Shao et al., 2025).

Emerging clinical data from China have provided additional insight into the potential role of this technique. Early studies and expert consensus reports suggest that prostatic urethral dilation may improve urinary symptoms in selected patients, including those with small prostate volume; however, the available evidence remains preliminary and should be interpreted with caution (Huang et al., 2025; Shao et al., 2025). Nevertheless, most currently available data originates from single-arm or observational studies, and differences in study design, patient selection, and follow-up duration should be considered when interpreting these findings. Given that the currently available PUD evidence remains preliminary, key outcomes such as IPSS, Qmax, PVR, adverse events, sexual or ejaculatory outcomes, and retreatment rates should be further evaluated in standardized prospective studies before robust outcome-level tabulation is attempted (Shao et al., 2025).

However, the potential limitations of PUD should be considered with the same caution applied to established therapies. Although the procedure is non-resective and non-ablative, perioperative and postoperative adverse events may still occur, including transient irritative urinary symptoms, dysuria, urinary retention, hematuria, urinary tract infection, pain, mucosal injury, and, less commonly, urethral trauma or stricture. The durability of symptom relief, retreatment rates, and long-term effects on sexual and ejaculatory function remain insufficiently defined. Patient selection is also critical, because outcomes may vary according to prostate size, bladder neck configuration, median lobe morphology, baseline bladder function, urethral compliance, and the presence of detrusor overactivity or detrusor underactivity. Patients with active urinary tract infection, urethral stricture, severe coagulopathy, suspected malignancy, or anatomical features unsuitable for catheter-based dilation may require alternative management. In addition, the procedure may be influenced by operator experience, device design, balloon positioning, dilation pressure, and local regulatory approval status. These unresolved issues should be considered before PUD is positioned within routine treatment algorithms (Huang et al., 2025; Shao et al., 2025).

Overall, PUD should be regarded as an emerging dilation-based option for selected patients with small-volume BPO rather than an established standard therapy. However, the current evidence base remains limited, and further multicenter randomized studies with long-term follow-up are needed to clarify its durability, comparative efficacy, and role within contemporary treatment algorithm (Huang et al., 2025; Shao et al., 2025).

To provide a more balanced overview, Table 1 summarizes the therapeutic principles, tissue effects, evidence status, and unresolved issues of PUD in relation to established surgical and minimally invasive therapies.

**TABLE 1:** Conceptual comparison of established surgical and minimally invasive therapies with prostatic urethral dilation for BPH/BPO.

Treatment	Main therapeutic principle	Main tissue or anatomical effect	Evidence status	Key limitations or unresolved issues
TURP	Endoscopic resection of obstructive prostatic tissue	Immediate tissue removal and enlargement of the prostatic urethral channel	Established surgical reference standard with long clinical experience	Perioperative bleeding, retrograde ejaculation, urethral stricture, urinary incontinence risk, and procedure-related morbidity
HoLEP	Laser enucleation of adenomatous prostatic tissue	Anatomical removal of obstructive adenoma with durable channel enlargement	Established surgical treatment with evidence supporting durable symptom relief	Learning curve, transient urinary incontinence, ejaculatory dysfunction, and perioperative considerations
PUL	Mechanical retraction of prostatic tissue using permanent implants	Implant-based widening of the prostatic urethral lumen without tissue resection	Clinical trial and real-world evidence available for selected patients	Anatomical suitability, median lobe considerations, implant-related issues, durability, and retreatment remain relevant considerations
Rezūm	Convective water vapor thermal therapy	Thermal injury followed by tissue necrosis, remodeling, and volume reduction	Clinical studies and follow-up data available for selected patients	Postoperative irritative symptoms, catheterization, delayed symptom improvement, urinary retention, infection, and retreatment may occur
iTIND	Temporary implantable nitinol device for mechanical remodeling of the prostatic urethra	Temporary mechanical remodeling without permanent tissue removal	Emerging clinical evidence	Device tolerance, patient selection, durability, retreatment, and long-term comparative effectiveness require further clarification
PAE	Selective embolization of prostatic arteries	Ischemic volume reduction and secondary tissue remodeling	Comparative and observational evidence available	Technical complexity, radiation exposure, vascular anatomical variation, variable outcomes, retreatment, and durability remain important concerns
PUD	Temporary circumferential dilation of the prostatic urethra	Non-resective and non-ablative enlargement of the urethral lumen; proposed reduction of urethral resistance	Early clinical reports and expert consensus; robust comparative evidence remains limited	Long-term durability, retreatment rates, adverse events, sexual and ejaculatory outcomes, patient selection, contraindications, learning curve, regulatory status, device dependence, and comparative effectiveness remain insufficiently defined

This conceptual comparison was informed by published studies, reviews, guidelines, and consensus reports on surgical and minimally invasive therapies for BPH/BPO, including TURP, HoLEP, PUL, Rezūm; iTIND, PAE, and PUD (McVary et al., 2011; Miernik and Gratzke, 2020; Abt et al., 2021; Couteau et al., 2021; Franco et al., 2023; Murad et al., 2023; Zhou et al., 2025; Hughes et al., 2023; Xu et al., 2025; Shao et al., 2025; Huang et al., 2025).

**Abbreviations:** BPH, benign prostatic hyperplasia; BPO, benign prostatic obstruction; HoLEP, holmium laser enucleation of the prostate; iTIND, temporarily implanted nitinol device; PAE, prostatic artery embolization; PUD, prostatic urethral dilation; PUL, prostatic urethral lift; TURP, transurethral resection of the prostate.

## 6 Discussion

Small-volume BPO represents a clinically important but still relatively under-recognized phenotype within the broader spectrum of male LUTS. The weak correlation between prostate size and symptom severity highlights the limitations of a purely volume-based model and suggests that functional and microenvironmental mechanisms may play a more substantial role than previously appreciated (Xu et al., 2025). This observation challenges the traditional view that anatomical enlargement alone is the primary determinant of bladder outlet obstruction.

At the same time, LUTS in patients with small prostate volume should not automatically be attributed solely to prostatic obstruction. Other conditions, including primary bladder neck obstruction, detrusor overactivity, reduced bladder compliance, detrusor underactivity, and dysfunctional voiding, overactive bladder, and neurogenic lower urinary tract dysfunction, may coexist with prostate-related obstruction or contribute to LUTS independently. Careful clinical evaluation therefore remains essential. In selected patients, urodynamic assessment may help separate bladder outlet obstruction from primary bladder dysfunction, especially when symptom severity is not proportional to prostate size or when treatment response is difficult to predict (Chess-Williams and Sellers, 2023; Abt et al., 2021; Papanikolaou et al., 2026). Careful clinical evaluation therefore remains essential. In addition to symptom assessment and imaging, urodynamic investigation may be helpful in selected patients to improve phenotypic classification and avoid inappropriate treatment selection.

The interaction among chronic inflammation, fibrosis, altered tissue biomechanics, and neural dysregulation provides a useful framework for understanding symptom heterogeneity in this population (Bushman and Jerde, 2016; de Groat et al., 2015). These mechanisms are unlikely to act independently. Inflammation may promote fibrotic remodeling, fibrosis may reduce urethral compliance, and neural dysregulation may amplify both smooth muscle tone and symptom perception. This integrated model helps explain why patients with similar prostate volumes may differ substantially in symptom severity, urinary flow, and treatment response (Murad et al., 2023; Xu et al., 2025).

This mechanism-based view also helps explain why treatment responses may vary considerably among patients with similar prostate volumes. Current pharmacological therapies mainly target smooth muscle tone, whereas surgical and minimally invasive procedures are largely designed to remove, ablate, or bypass obstructive tissue (Farmer and Noble, 1997). As a result, important contributors such as fibrosis, inflammation, and neural dysfunction may remain insufficiently addressed. This mismatch between disease mechanisms and therapeutic targets may be particularly relevant in small-volume BPO, where functional obstruction appears to predominate (Farmer and Noble, 1997).

Within this context, PUD represents a mechanistically plausible but still incompletely validated treatment approach. By reducing urethral resistance through controlled mechanical expansion without tissue destruction, the procedure may address one component of outlet resistance while preserving tissue integrity. Compared with conventional resective surgery, this non-ablative strategy may reduce iatrogenic injury and may help preserve sexual and continence function, which have become increasingly important considerations in contemporary patient-centered care (Couteau et al., 2021). In addition, the relatively straightforward procedural workflow and limited dependence on highly complex equipment may improve accessibility across different clinical settings, although this practical consideration requires further evaluation.

Compared with other minimally invasive therapies, PUD also occupies a somewhat distinct conceptual position. Procedures such as Rezūm and PAE rely on tissue necrosis and subsequent remodeling, whereas PUL primarily achieves symptom relief through mechanical tissue retraction (Zhou et al., 2025). In contrast, PUD attempts to modify urethral resistance while preserving tissue viability. This procedural distinction may be relevant in patients with small prostate volume, in whom excessive tissue bulk is not the dominant pathological feature. However, whether this theoretical advantage ultimately translates into superior long-term clinical outcomes remains uncertain.

Early clinical studies, including emerging reports from China, suggest that PUD may improve urinary symptoms and urinary flow in selected patients, although the available evidence remains preliminary (Shao et al., 2025; Huang et al., 2025). Nevertheless, the currently available evidence remains limited. Most studies are observational in nature, involve relatively small sample sizes, and differ considerably in patient selection, procedural technique, and duration of follow-up. Consequently, the long-term durability of symptom improvement, retreatment rates, and potential late complications remain insufficiently defined. Future translational studies should examine whether dilation is associated with measurable changes in urethral compliance, periurethral matrix organization, smooth muscle responsiveness, mucosal integrity, or local sensory signaling, because these mechanisms remain largely inferential at present.

Thus, current clinical observations support only the possibility of symptomatic and uroflow improvement after PUD; they do not yet establish that dilation durably modifies tissue compliance, fibrotic remodeling, mucosal injury responses, inflammatory activity, or neural regulation.

Several issues deserve further investigation. Well-designed multicenter randomized controlled trials are needed to compare PUD with established surgical and minimally invasive therapies. More refined patient selection strategies based on urodynamic findings, imaging characteristics, and possibly molecular or biomarker profiling may also improve treatment outcomes. In addition, future studies should evaluate long-term efficacy, durability, and functional preservation in a standardized manner. Combination approaches integrating PUD with pharmacological therapy may represent another potential direction, particularly for patients with both functional and structural contributors to obstruction.

Overall, PUD should be regarded as an emerging dilation-based option for selected patients with small-volume BPO. However, its precise role within contemporary management pathways remains to be clarified. A gradual shift toward mechanism-based and phenotype-oriented treatment strategies may ultimately allow more individualized and effective management of male LUTS beyond prostate size alone.

## 7 Conclusion

Small-volume BPO appears to represent a clinically relevant phenotype in which functional mechanisms may play a greater role than gland size alone, although anatomical factors may still coexist in selected patients (Mostwin et al., 2005). Multiple mechanisms, including increased smooth muscle tone, chronic inflammation, fibrotic remodeling, altered tissue biomechanics, and neural dysregulation, likely interact to drive symptom development and bladder outlet resistance (Chess-Williams and Sellers, 2023). This perspective highlights the limitations of relying solely on prostate size when evaluating disease severity and selecting treatment strategies (Xu et al., 2025).

Within this setting, ultra-minimally invasive prostatic urethral dilation (PUD), as defined in this review as a non-resective and non-ablative dilation-based procedure, may be considered a potential option for carefully selected patients. By reducing urethral resistance through controlled mechanical expansion without tissue destruction, PUD may be relevant for selected patients, particularly those with small prostate volume and a desire to preserve sexual and continence function (Couteau et al., 2021). Its proposed mechanical rationale differs from conventional tissue-reductive or ablative therapies. Nevertheless, PUD should be regarded as an emerging treatment option rather than an established replacement for standard medical, surgical, or minimally invasive therapies, and its current clinical role remains insufficiently validated.

At present, however, the available evidence remains limited. Most published data are derived from early clinical experience and single-arm or observational studies, and important questions regarding long-term durability, retreatment rates, safety, patient selection, and comparative effectiveness remain unanswered. Larger multicenter randomized controlled studies with standardized outcome assessment and longer follow-up are therefore needed to better define the role of PUD within current treatment pathways.

More broadly, growing recognition of functional and microenvironmental mechanisms in small-volume BPO may support a gradual shift toward phenotype-oriented and mechanism-based management strategies. Such an approach may ultimately allow more individualized and clinically relevant treatment selection for men with LUTS beyond prostate size alone.

## References

[B1] Abrams P. Cardozo L. Fall M. Griffiths D. Rosier P. Ulmsten U. (2002). The standardisation of terminology of lower urinary tract function: report from the standardisation Sub-committee of the international continence society. Neurourol. Urodyn. 21, 167–178. 10.1002/nau.10052 11857671

[B2] Abt D. Müllhaupt G. Hechelhammer L. Markart S. Güsewell S. Schmid H. P. (2021). Prostatic artery embolisation versus transurethral resection of the prostate for benign prostatic hyperplasia: 2-yr outcomes of a randomised, open-label, single-centre trial. Eur. Urol. 80, 34–42. 10.1016/j.eururo.2021.02.008 33612376

[B3] Bushman W. A. Jerde T. J. (2016). The role of prostate inflammation and fibrosis in lower urinary tract symptoms. Am. J. Physiol. Renal Physiol. 311, F817–F821. 10.1152/ajprenal.00602.2015 27440781

[B4] Chen J. Sun W. Zhu Y. Zhao F. Deng S. Tian M. (2024). TRPV1: the key bridge in neuroimmune interactions. J. Intensive Med. 4, 442–452. 10.1016/j.jointm.2024.01.008 39310069 PMC11411435

[B5] Chess-Williams R. Sellers D. J. (2023). Pathophysiological mechanisms involved in overactive bladder/detrusor overactivity. Curr. Bladder Dysfunct. Rep. 18, 79–88. 10.1007/s11884-023-00690-x

[B6] Cornu J. Gacci M. Hashim H. Herrmann T. Malde S. Netsch C. (2023). “EAU guidelines on management of non-neurogenic male LUTS. EAU guidelines,” in Presented at the EAU Annual Congress Milan.

[B7] Couteau N. Duquesne I. Panthier F. Thiounn N. Timsit M.-O. Mejean A. (2021). Ejaculations and benign prostatic hyperplasia: an impossible compromise? A comprehensive review. J. Clin. Med. 10, 5788. 10.3390/jcm10245788 34945084 PMC8704358

[B8] Das A. K. Han T. M. Uhr A. Roehrborn C. G. (2020). Benign prostatic hyperplasia: an update on minimally invasive therapy including Aquablation. Can. J. Urol. 27, 2–10. 32875996

[B9] De Groat W. C. Griffiths D. Yoshimura N. (2015). Neural control of the lower urinary tract. Compr. Physiol. 5, 327–396. 10.1002/cphy.c130056 25589273 PMC4480926

[B10] Devesa I. Ferrándiz-Huertas C. Mathivanan S. Wolf C. Luján R. Changeux J.-P. (2014). “αCGRP is essential for algesic exocytotic mobilization of TRPV1 channels in peptidergic nociceptors,” in Proceedings of the National Academy of Sciences, 111, 18345–18350. 10.1073/pnas.1420252111 25489075 PMC4280602

[B11] Elliott C. S. Payne C. K. (2012). “Evaluation and treatment of Male lower urinary tract symptoms,” in Essential Urology: A Guide to Clinical Practice. Editor Potts J. M. (Totowa, NJ: Humana Press).

[B12] European association of urology (2026). EAU guidelines on the management of non-neurogenic Male LUTS. Edn. presented at the EAU Annual Congress London 2026 ed. Arnhem, The Netherlands.

[B13] Farmer A. Noble J. (1997). Drug treatment for benign prostatic hyperplasia: decreasing muscle tone within the prostate gland may be more effective than reducing the size of the prostate. BMJ 314, 1215–1216. 10.1136/bmj.314.7089.1215 9154021 PMC2126611

[B14] Fibbi B. Penna G. Morelli A. Adorini L. Maggi M. (2010). Chronic inflammation in the pathogenesis of benign prostatic hyperplasia. Int. J. Androl. 33, 475–488. 10.1111/j.1365-2605.2009.00972.x 19508330

[B15] Fowler C. J. Griffiths D. De Groat W. C. (2008). The neural control of micturition. Nat. Rev. Neurosci. 9, 453–466. 10.1038/nrn2401 18490916 PMC2897743

[B16] Franco J. V. A. Tesolin P. Jung J. H. (2023). Update on the management of benign prostatic hyperplasia and the role of minimally invasive procedures. Prostate Int. 11, 1–7. 10.1016/j.prnil.2023.01.002 36910900 PMC9995694

[B17] Grant M. J. Booth A. (2009). A typology of reviews: an analysis of 14 review types and associated methodologies. Health Info Libr. J. 26, 91–108. 10.1111/j.1471-1842.2009.00848.x 19490148

[B18] Green B. N. Johnson C. D. Adams A. (2006). Writing narrative literature reviews for peer-reviewed journals: secrets of the trade. J. Chiropr. Med. 5, 101–117. 10.1016/S0899-3467(07)60142-6 19674681 PMC2647067

[B19] Huang B. Kang J. Yuan Q. Liu L. (2025). Chinese expert consensus on the treatment of benign prostatic hyperplasia by prostatic urethral dilation. J. Clin. Urol. 40 (12), 1027–1032. 10.13201/j.issn.1001-1420.2025.12.001

[B20] Hughes T. Harper P. Somani B. K. (2023). Treatment algorithm for management of benign prostatic obstruction: an overview of current techniques. Life 13, 2077. 10.3390/life13102077 37895457 PMC10608556

[B21] Kaltsas A. Giannakas T. Stavropoulos M. Kratiras Z. Chrisofos M. (2025). Oxidative stress in benign prostatic hyperplasia: mechanisms, clinical relevance and therapeutic perspectives. Diseases 13, 53. 10.3390/diseases13020053 39997060 PMC11854834

[B22] Khan A. Alzahrani H. A. Felemban S. G. Algarni A. S. Alenezi A. B. S. Kamal M. (2025). Exploring TGF-β signaling in benign prostatic hyperplasia: from cellular senescence to fibrosis and therapeutic implications. Biogerontology 26, 79. 10.1007/s10522-025-10226-x 40159577

[B23] Li T. Wang G. Hui V. C. C. Saad D. De Sousa Valente J. La Montanara P. (2021). TRPV1 feed-forward sensitisation depends on COX2 upregulation in primary sensory neurons. Sci. Rep. 11, 3514. 10.1038/s41598-021-82829-6 33568699 PMC7876133

[B24] Mcvary K. T. Roehrborn C. G. Avins A. L. Barry M. J. Bruskewitz R. C. Donnell R. F. (2011). Update on AUA guideline on the management of benign prostatic hyperplasia. J. Urology 185, 1793–1803. 10.1016/j.juro.2011.01.074 21420124

[B25] Miernik A. Gratzke C. (2020). Current treatment for benign prostatic hyperplasia. Dtsch. Arztebl Int. 117, 843–854. 10.3238/arztebl.2020.0843 33593479 PMC8021971

[B26] Mostwin J. Bourcier A. Haab F. Koebl S. Rao N. Resnick S. (2005). Pathophysiology of Urinary Incontinence, Fecal Incontinence and Pelvic Organ Prolapse in Incontinence. 3nd ed. (Plymouth: Health Publication Ltd), 1, 436–462.

[B27] Murad L. Bouhadana D. Nguyen D. D. Chughtai B. Zorn K. C. Bhojani N. (2023). Treating LUTS in men with benign prostatic obstruction: a review article. Drugs Aging 40, 815–836. 10.1007/s40266-023-01054-0 37556075

[B28] Papanikolaou D. Diamantopoulos C. Sokolakis I. Kolvatzis M. Antoniadis G. Moysidis K. (2026). The role of the aging bladder in lower urinary tract symptoms: pathophysiology and therapeutic implications in patients with benign prostatic hyperplasia. Medicina 62, 685. 10.3390/medicina62040685 42075556 PMC13117785

[B29] Plochocki A. King B. (2022). Medical treatment of benign prostatic hyperplasia. Urol. Clin. 49, 231–238. 10.1016/j.ucl.2021.12.003 35428429

[B30] Ren J. Li Y. Zhang X. Xiong M. Zhang H. An L. (2024). Correlation between metabolic syndrome and periurethral prostatic fibrosis: results of a prospective study. BMC Urol. 24, 38. 10.1186/s12894-024-01413-y 38347470 PMC10863095

[B31] Roehrborn C. G. (2005). Benign prostatic hyperplasia: an overview. Rev. Urol. 7 (Suppl. 9), S3–S14. 16985902 PMC1477638

[B32] Savvides E. Langas G. Sountoulides P. (2024). Editorial for special issue: current and emerging therapies for lower urinary tract symptoms and benign prostatic hyperplasia. J. Clin. Med. 13, 2453. 10.3390/jcm13092453 38730982 PMC11084587

[B33] Shao N. Cao Q. Cui X. Chen C. Shen K. Kang J. (2025). Technical advances and evaluation of safety and efficacy in super minimally invasive prostatic urethral dilation surgery. J. Clin. Urol. 40 (9), 811–817. 10.13201/j.issn.1001-1420.2025.09.010

[B34] Xu J. Han B. Xia S. Jing Y. (2025). Beyond size: a comprehensive overview of small-volume benign prostatic hyperplasia. Curr. Urol. 19, 1–5. 10.1097/cu9.0000000000000261 40313426 PMC12042193

[B35] Yoosuf B. T. Panda A. K. Kt M. F. Bharti S. K. Devana S. K. Bansal D. (2024). Comparative efficacy and safety of alpha-blockers as monotherapy for benign prostatic hyperplasia: a systematic review and network meta-analysis. Sci. Rep. 14, 11116. 10.1038/s41598-024-61977-5 38750153 PMC11096304

[B36] Zhou Y. Luo Q. Wang R. Zheng D. Xiong Y. Zuo J. (2025). Integrated management strategies for benign prostatic hyperplasia. Front. Urology 5, 1641171. 10.3389/fruro.2025.1641171 41158964 PMC12554669

